# Host Gender and Androgen Levels Regulate Gut Bacterial Taxa in Pigs Leading to Sex-Biased Serum Metabolite Profiles

**DOI:** 10.3389/fmicb.2019.01359

**Published:** 2019-06-18

**Authors:** Maozhang He, Jun Gao, Jinyuan Wu, Yunyan Zhou, Hao Fu, Shanlin Ke, Hui Yang, Congying Chen, Lusheng Huang

**Affiliations:** State Key Laboratory of Pig Genetic Improvement and Production Technology, Jiangxi Agricultural University, Nanchang, China

**Keywords:** sex bias, gut microbiota, 16S rRNA gene, serum metabolome, swine

## Abstract

Gut microbiota regulates host metabolism and immunity. The phylogenetic composition of gut microbiota is influenced by diverse factors that include host gender. In this study, the effects of gender on gut microbial composition and its subsequent influence on serum metabolites in pigs were evaluated. The bacterial composition of feces samples was determined by 16S rRNA gene sequencing in 293 pure-bred Duroc pigs (108 gilts and 185 entire boars) and 64 validated pigs from an eight-breed mosaic F_6_ population. Twenty-eight F_6_ boars were castrated at 80 days of age to evaluate the effects of androgen on gut microbial composition. Untargeted serum metabolite features were determined in 45 boars and 26 gilts by an ultra-performance liquid chromatography coupled with quadrupole time-of-flight mass spectrometry (UPLC-QTOF/MS). The study observed an obvious influence of host gender on the gut microbial composition and identified numerous sex-biased bacterial taxa. These included Veillonellaceae, *Roseburia*, *Bulleidia*, and *Escherichia* which showed the higher abundance in boars, and *Treponema* and *Bacteroides* which were over-represented in gilts. Castration significantly shifted the fecal microbiota composition of the boars toward that of gilts. The predicted functional pathways of the gut microbiome related to obesity and energy harvest were enriched in gilts, and positively associated with gilt-enriched bacteria. Functional pathways related to peptidases and carbohydrate metabolism were enriched in boars and positively associated with boar-enriched bacteria. Serum metabolites related to androgen and cresol metabolism were identified as sex-biased metabolites. Correlation analysis between serum metabolites and sex-biased bacteria identified that the serum concentration of androgen-related metabolites was positively correlated with *Bulleidia* and *Escherichia*, but negatively associated with *Treponema*, suggesting a significant interaction between gut microbiota and host sex hormone metabolism. These results offer basic knowledge of how host gender and gut microbiota influence host metabolism.

## Introduction

The gut microbial community is an intricate and heterogeneous ecosystem that is influenced by the environment, diet, and the host (Wu et al., 2011; David et al., 2014; Goodrich et al., 2014; Tong et al., 2014; Org et al., 2015; Benson, 2016; Bonder et al., 2016; Singh et al., 2017). In recent years, an increasing number of studies have indicated profound interactions between host gender and the gut microbiome including the influence of puberty in male mice, which is less diverse than that of their female counterparts (Yurkovetskiy et al., 2013). In comparison to normal males, sexually mature castrated male mice have a similar gut microbial composition to females, demonstrating the influence of androgens on gut microbial composition (Markle et al., 2013; Yurkovetskiy et al., 2013). Furthermore, several reports highlight the impact of gut microbiota on disease, including cardiometabolic disorders and type 1 diabetes, mediated through its effects on sex hormones (Kudelka et al., 2016; Xie et al., 2017). Sex biases in the gut microbiome drive the hormonal-dependent regulation of autoimmunity (Markle et al., 2013). Emerging evidence also suggests that sex hormones regulate host immune function through the activation of specific gut microbe-associated toll like receptors and NOD like receptors (Rizzetto et al., 2018). A bi-directional interaction between gut microbiota and sex hormones has been demonstrated. Host hormones impact gut bacterial gene expression and composition, whilst the gut microbiota influences the levels of sex hormones. For example, *Clostridium scindens* can convert glucocorticoids to androgens (Ridlon et al., 2013). In addition, the effect of diet on the gut microbiota is now known to be sex-dependent (Bolnick et al., 2014; Org et al., 2016). In pigs, host gender significantly influences the phylogenetic composition of the gut bacterial community (Xiao et al., 2016). Entire male pigs have a higher concentration of skatole than gilts (Zhou et al., 2015). The microbial degradation of tryptophan in the hindgut of the pigs produces skatole. Several studies have shown that the bacteria participating in the production of skatole and indole are gender-related, including *Clostridium*, *Bacteroides*, and *Lactobacillus* (Jensen et al., 1995; Wesoly and Weiler, 2012). Despite this knowledge, systematical studies on the influence of sex hormones on gut microbes are lacking in the literature.

Sex differences in the gut microbiome drive host metabolism and influences the serum metabolite profile (Markle et al., 2013; Xie et al., 2017). Several studies have explored variations in metabolic molecules in biofluid samples (serum and urine) between genders. Mittelstrass et al. (2011) identified significant gender-biases in the abundance of amino acids, lipids, and sugar in the serum. Krumsiek et al. (2015) found that nearly one-third of the total metabolome significant differs between female and male subjects, and that the metabolites produced from steroid, fatty acid and amino acid metabolism show strong differences across genders. However, the relationship between sex-biased gut microbiota and serum metabolites remains largely uncharacterized.

In this study, 16S rRNA gene sequencing was performed for fecal samples and untargeted metabolomic profiles of host serum samples were measured to comprehensively characterize sexual differences in porcine fecal microbiota composition and serum metabolites. Bacterial taxa and predicted KEGG pathways in the gut microbiome that were significantly influenced by host gender were identified. Furthermore, a subset of serum metabolites showing differential abundances between boars and gilts were detected. The relationship between sex-dependent bacteria and metabolites were further established herein.

## Materials and Methods

### Animals and Fecal Samples

Two experimental pig cohorts were studied including 293 commercial pure-breed Duroc pigs (185 entire boars and 108 gilts) from Jiangyin farm as the discovery cohort and 64 F_6_ pigs from a heterogeneous intercross as the validated cohort. The experimental pigs of the discovery cohort were raised and managed as described in our previous study (Yang et al., 2017). Briefly, all pigs were raised in uniformed farm conditions and provided the same commercial formula diet and clean water *ad libitum*. Fecal samples were collected from each animal’s anus at 140 days of age. The validated pig cohort was raised in an experimental pig farm of the state key laboratory of pig genetic improvement and production technology (Jiangxi province, China). Pigs were provided the same formula diet twice a day. Water was available *ad libitum* from nipple drinkers. Fecal samples from 28 boars were collected at 80 days of age before castration (entire boars) and 120 days of age after castration. As the counterparts, fecal samples from 36 entire gilts were also harvested at both 80 and 120 days of age. All fecal samples were immersed in liquid nitrogen immediately after collection, and stored at −80°C until use. All experimental pigs were healthy and did not receive any other antibiotic treatment within 2 months of fecal sample collection.

### Ethics Statement

All animal work was conducted according to the guidelines for the care and use of experimental animals established by the State Council of the People’s Republic of China (Decree No. 2, 1988). This study was also approved by Animal Care and Use Committee (ACUC) in Jiangxi Agricultural University (No. JXAU2011-006).

### High-Throughput Sequencing of the Bacterial 16S rRNA Gene

Microbial DNA was extracted from fecal samples using QIAamp Fast DNA Stool Mini Kits (Qiagen, Germany) according to the manufacturer’s protocol (McOrist et al., 2002). DNA concentration and integrity were measured on a Nanodrop-1000 and through 0.8% agarose gel electrophoresis. The fusion primers 515F (5′-GTGCCAGCMGCCGCGGTAA) and 806R (5′-GGACTACHVGGGTWTCTAAT) with dual index were used for amplifying the V4 region of the bacterial 16S rRNA gene under a melting temperature of 60°C with 30 cycles. Amplicon sequencing was performed on an Illumina MiSeq platform (Illumina, United States). Paired-end reads from the clean data sets were assembled into tags using FLASH (v.1.2.11) (Magoc and Salzberg, 2011). To avoid statistical bias caused by an uneven sequencing depth, each sample sequences was rarefied to 16,000 tags in all experimental pigs. Tags were clustered into operational taxonomic units (OTUs) of 97% similarity using USEARCH software (v7.0.1090) (Majaneva et al., 2015) and the UPARSE-OTU algorithm. OTU taxonomic assignments for the 16S rRNA sequences were produced using the RDP classifier program (v2.2) (Wang et al., 2007). The α-diversity indexes including observed species and Shannon were measured using mothur software (Schloss et al., 2009).

### Determination of the Metabolomic Profiles of Porcine Serum Samples

Seventy-one serum samples from the Duroc pig cohort (45 entire boars and 26 gilts) were used for untargeted metabolomic analysis by an ultra-performance liquid chromatography coupled with quadrupole time-of-flight mass spectrometry (UPLC-QTOF/MS). All serum samples were thawed on ice and precipitated with pre-cooled methanol (Merck Corp., Germany) as previously shown (Liu et al., 2017a). Briefly, 300 μl of cooled methanol was added to 100 μl serum, which was vortexed for 1 min, incubated at −20°C for 20 min, and centrifuged at 15,000 × *g* (rcf) for 15 min at 4°C. Supernatants were removed into clean tubes and dried in a Savant vacuum evaporator. Dried supernatants were resolved in 150 μL water: methanol (85%:15% v/v) and transferred into the sampling vials pending UPLC-QTOF/MS (Waters Corp., United States) analysis (Dunn et al., 2011). In addition, the pooled quality control (QC) sample was prepared by combining aliquots of equal volume for each tested sample.

Working solution (1.0 μL) was injected into a 100 mm × 2.1 mm, 1.7 μm BEH C18 column (Waters Corp., United States). The pooled QC sample was injected eight times at the beginning of the run to ensure system equilibrium, and then one time for each of twelve samples to further monitor analytical stability. For the positive electrospray ion mode (ES^+^), serum samples were eluted using a linear gradient from 100% A to 100% B (A, water + 0.1% formic acid; B, acetonitrile) at a flow rate of 0.3 mL/min and a column temperature of 40°C for 22 min. For the negative electrospray ion mode (ES^−^), injected serum samples were eluted on a linear gradient of 100% A to 100% B (A, water + 0.1% formic acid; B, acetonitrile) at a flow rate of 0.3 mL/min at 40°C of the column temperature for 18 min.

Mass spectrometric data was collected using a Waters Q-TOF Premier (Waters Corp., United States) equipped with an electrospray source operating in either ES^+^ or ES^−^. The source temperature was set at 120°C, and the desolvation gas temperature was set at 350°C. The capillary voltage was set at 3.0 and 2.5 kV for ES^+^ and ES^−^, respectively. Centroid data were collected from 50 to 1200 m/z with a scan time of 0.3 s and interscan delay of 0.02 s. MassLynx software (Waters Corp., United States) was used for system control and data acquisition. Leucine enkephalin was used as the lock mass (m/z 556.2771 in ES^+^ and 554.2615 in ES^−^) at a concentration of 100 ng/mL and a flow rate of 5 μL/min for all analyses. Progenesis QI software (v2.0) (Nonlinear Dynamics, United Kingdom) was used for feature alignment, non-targeted signal detection and signal integration.

MetaScope embedded in the Progenesis QI (Rusilowicz et al., 2016) was used to annotate the metabolites not only based on neutral mass, isotope distribution and retention time, but also based on the collisional cross-sectional area and MS/MS fragmentation data in the HMDB database. Ion intensity of each peak was obtained and a 3D-matrix containing arbitrarily assigned peak indices (retention time-m/z pairs), ion intensities (variables) and sample names (observations) was generated. Raw matrices were further filtered by removing peaks with missing values (ion intensity = 0) in more than 80% of the samples and 50% of the QC samples. Each retained peak was then normalized to the QC sample using support vector regression (SVR) from the R package MetNormalizer (Shen et al., 2016) to ensure a high quality of data within an analytical run. The relative standard deviation (RSD) values of the metabolites in the QC samples was set at a threshold of 30% to assess the repeatability of metabolomic data sets.

### Statistical Analysis

Bray–Curtis distances were calculated based on the OTU data using R package vegan. Principle coordinate analysis (PCoA) was performed to highlight the discrepancy of the phylogenetic compositions of gut microbiota between boars and sows, or between entire and castrated boars using the ade4 in R package. An analysis of similarities (ANOSIM) was used to assess the differences in gut microbiota amongst the groups using the R package vegan. An R value close to 0 represents no significant difference between differentiations of inter- and intra-groups. An R value close to 1 represents that inter-group differentiation was greater than intra-group differences. *P* < 0.05 reflects a statistical significance. To predict the functional capacity of the gut microbiome, PICRUST (v1.0.0) software was used to predict the functional capacities of gut microbiome (KEGG Orthologies) from 16S rRNA gene sequencing data against Greengenes database. And then, the predicted KEGG Orthologies were classified into each KEGG pathway. The relative abundance of KEGG pathways was then calculated (Langille et al., 2013). Linear discriminant analysis effect size (LEfSe) analysis was used to identify sex-biased KEGG pathways using standard parameters (*P* < 0.05 and LDA score >2.0). A Wilcoxon rank-sum test was used for the comparison of gut microbial composition that did not fit the normal distribution between two groups. Raw *P*-values were adjusted for multiple testing using the Benjamini–Hochberg method at a false discovery rate (FDR) of 0.05 (Benjamini and Hochberg, 1995). Sparse Partial Least Squares-Discriminant analysis (sPLS-DA) was performed to evaluate the sex bias of the untargeted metabolome by MetaboAnalyst^1^ (Xia and Wishart, 2016). A Wilcoxon *t*-test was used to identify sex-biased metabolites at the significance threshold of adjusted *P*-values ≤0.2. Spearman rank correlation was used to assess the relationship between sex-biased bacterial taxa and serum metabolites or predicted KEGG pathways of the gut microbiome. Multiple testing was adjusted using the Benjamini–Hochberg method. Scatter plots and heatmaps were plotted using the ggpubr and ggplot2 package in R software.

To determine the OTUs that could be used to distinguish entire boars, gilts and castrated boars, random-forest models with modified settings were constructed (ntree = 1000). The optimal number of OTUs was determined by 10-fold cross-validation using the rfcv function of random Forest package. The most highly discriminating OTUs were identified by importance values characterized by “MeanDecreaseAccuracy” parameters. The interpolated area under the receiver operating characteristic (ROC) curve (AUC) was determined to evaluate the diagnostic accuracy of the model (R 3.3.2; pROC3 package) (Breiman, 2001; Andy Liaw, 2002).

### Construction of Serum Metabolite Feature Modules

Metabolite features were marked as endogenic metabolites according to the HMDB database and used to construct the metabolite feature modules. Metabolite datasets were first normalized by log_10_ transformation of the m/z values, and used to construct the modules via soft-threshold Pearson correlation analysis in combination with a topological overlap distance metric and average hierarchical clustering [weighted correlation network analysis (WGCNA) in the R package] (Langfelder and Horvath, 2008). Briefly, a signed network and soft threshold of 3 were used to satisfy the scale free topology criteria. The parameters (deepSplit = 4 and minModuleSize = 10) embedded in the dynamic tree cut function were established (Langfelder et al., 2008). The eigenmetabolites were defined as the first singular vector of each sample and used to calculate the Pearson correlation coefficient between the metabolic modules and the relative abundance of sex-biased bacteria. Significant correlations were determined using Student asymptotic *P*-values. Visualization of the network was performed using the gplot function in the sna of R package.

## Results

### Host Gender Influences the Phylogenetic Composition of Gut Microbiota

**FIGURE 1:**
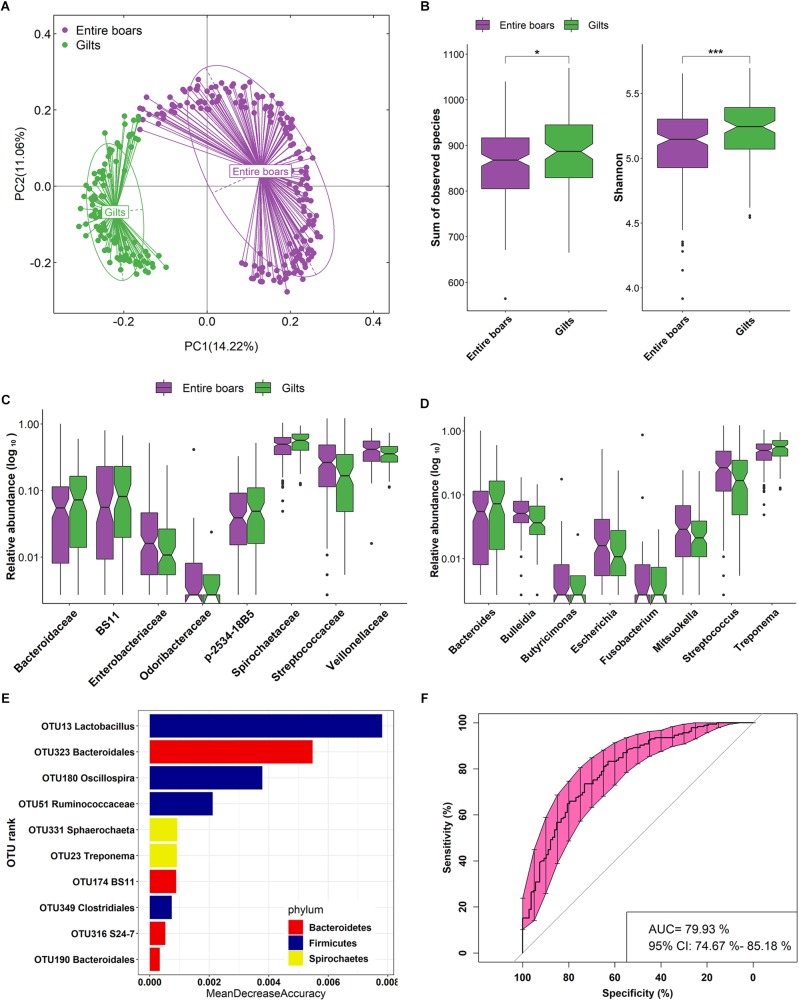
Distinct gut microbial compositions between entire boars and gilts in the Duroc pig cohort. **(A)** Distinct gut microbial compositions identified by Principle coordinates analysis which was performed based on Bray–Curtis distance. **(B)** Comparison of α-diversity of the fecal microbiota (observed species and Shannon index) between gilts and entire boars. Violin plots show that gilts had the higher richness and diversity of fecal microbiota than entire boars. ^∗^*P* < 0.05, ^∗∗^*P* < 0.01, and ^∗∗∗^*P* < 0.001. **(C,D)** The sex-biased bacterial taxa at the family and genus level. **(E)** The top 10 biomarkers of OTUs that could discriminate the male and female samples by Random Forest model. Biomarker OTUs were ranked in descending order of importance to the accuracy of the model. **(F)** Receiver operating curve (ROC) for the Duroc population. The AUC was 79.93% with the 95% CI of 74.67–85.18%.

After quality control, an average of 32,457 high quality tags for each sample was obtained in Duroc pigs. Based on a 97% sequence similarity, an average of 896 OTUs per sample was detected. At the taxonomic level and consistent with previous reports in pigs (Isaacson and Kim, 2012; Looft et al., 2014), the phylogenetic composition of the fecal microbial community was dominated by Firmicutes (45.36%), Bacteroidetes (42.15%), Proteobacteria (4.10%), and Spirochaetes (3.35%). An obvious global shift in the fecal bacterial composition was detected between gilts and entire boars according to PCoA and ANOSIM analysis (*R* = 0.659, *P* = 1.00E-03, Figure 1A). The α-diversity of the fecal microbiota of gilts was significantly higher than that of entire boars with respect to observed species and Shannon indices (*P* = 0.04 and 9.82E-04, respectively; Figure 1B).

To decipher the bacterial taxa influenced by host gender, the relative abundance of bacterial taxa was compared between entire boars and gilts using Wilcoxon rank-sum tests. At the family level, eight families were identified with significantly different abundances between entire boars and gilts in the Duroc population. Veillonellaceae and Enterobacteriaceae were more abundant in entire boars (*P* = 7.80E-03 and 7.40E-03, respectively). However, Spirochaetaceae and Bacteroidaceae were significantly enriched in gilts (*P* = 7.20E-03 and 0.01, respectively) (Figure 1C). At the genus level, a total of 8 bacterial genera displayed differing enrichments between entire boars and gilts (Figure 1D). For example, *Bulleidia* and *Escherichia* were of higher abundance in entire boars, whilst *Treponema* and *Bacteroides* were significantly enriched in gilts. At the OTU level, 49 OTUs were identified with distinct abundances between entire boars and gilts. These sex-biased OTUs were mainly annotated to Ruminococcaceae, Lachnospiraceae, *Lactobacillus*, *Coprococcus*, *Oscillospira*, *Treponema*, *Ruminococcus*, *Streptococcus*, and *Prevotella* (Supplementary Table S1). Subsequently, a Random Forest (RF) analysis was performed to examine our ability to discriminate samples from boars or gilts based on the fecal microbiota composition. The results showed that 10 OTUs could distinguish male and female samples with robust and high diagnostic accuracy of the area under the curve (AUC) 79.93% (Figure 1E,F). These OTUs were annotated to the taxa including BS11 and *Treponema*, identified as sex-biased bacteria at the taxonomical level.

### Validating the Influence of Host Gender on the Gut Microbial Composition in an Independent Pig Cohort

**FIGURE 2:**
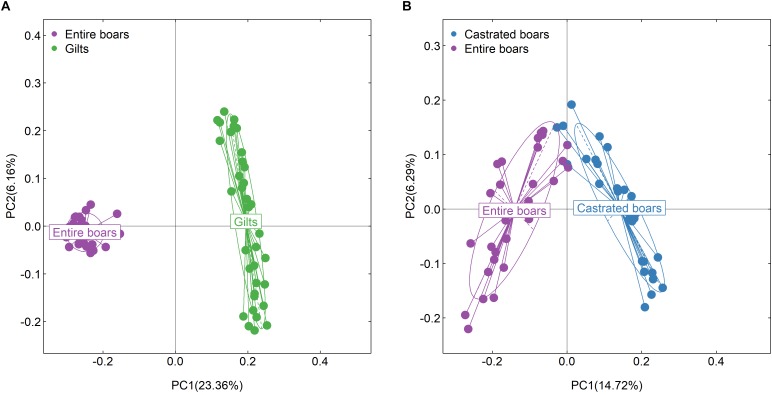
Validation of the host gender effect on fecal microbial composition, and the shifts of boar fecal microbiota composition before and after castration in the F_6_ cohort. **(A)** PCoA plot based on relative abundance of OTUs showing significant difference of gut microbial composition between entire boars and gilts in the F_6_ population. **(B)** PCoA analysis indicates the significant difference of fecal microbial composition before and after castration. Each point represents the fecal microbiota of a sample.

To further confirm the impact of host gender on the gut microbial composition, fecal samples from 28 entire boars and 36 gilt counterparts in the F_6_ pig cohort were performed by 16S rRNA gene sequencing. The phylogenetic composition of gut microbiota at the phylum level was in concordance with that in the Duroc population. Through PCoA analysis, a statistically significant separation of gut microbiota between samples from entire boars and gilts was also observed (ANOSIM, *R* = 0.995, *P* = 1.00E-03) (Figure 2A). Furthermore, in comparison to entire boars, both Observed species and Shannon indices were significantly higher in gilts (*P* = 4.10E-03 and 2.10E-06, respectively, Supplementary Figure S1A).

At the taxonomic level, 18 families and 16 genera were identified with significantly distinct abundance between entire boars and gilts (Supplementary Figures S1B,C). Amongst these, six families and three genera of sex-biased bacteria were observed in both experimental populations, including BS11, p-2534-18B5, Spirochaetaceae, and the predominant genus *Treponema*, all of which were enriched in gilts. A total of 128 OTUs displayed differential abundances between entire boars and gilts, including 70 OTUs annotated to Ruminococcaceae, Lachnospiraceae, *Prevotella*, *Coprococcus*, *Oscillospira*, *Treponema*, *Faecalibacterium*, and *Ruminococcus*, also identified as the sex-biased taxa in Duroc pigs (Supplementary Table S2). Random forest classification analysis was used to further identify bacteria that can accurately distinguish entire boars from gilts. The 10 most discriminative OTUs could distinguish gilts from entire boars with an accuracy of AUC 94.54%. OTU8, OTU1424, and OTU12 were annotated to *Treponema* which was identified as a robust sex-biased bacterial marker in two pig cohorts (Supplementary Figures 1D,E).

### Evaluating the Effect of Androgens on the Fecal Microbiota Composition by Gonadectomy

**FIGURE 3:**
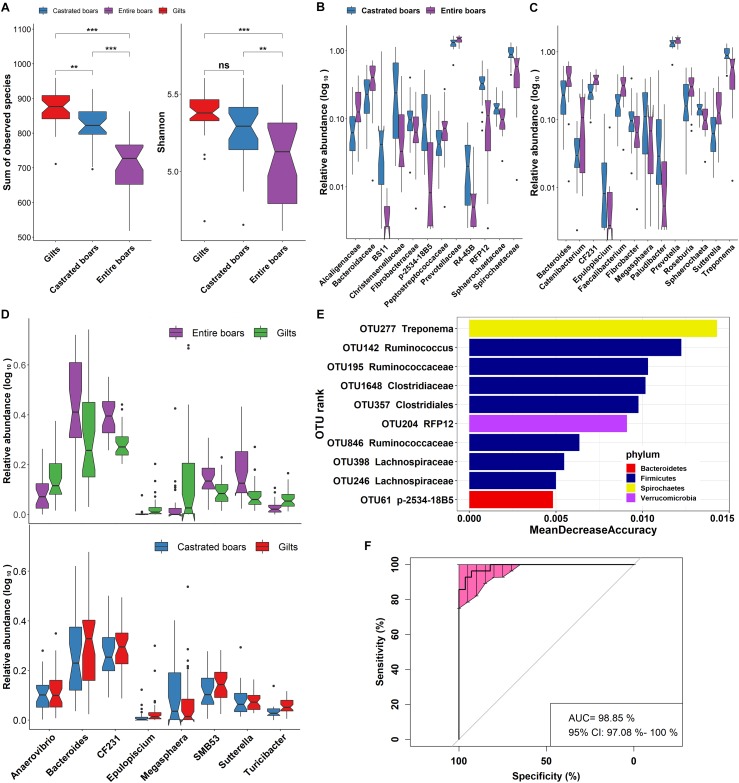
Significant shifts of gut microbial compositions before and after castration. **(A)** Comparison of feces microbial compositions among castrated boars, entire boars, and gilts based on the index of observed species and Shannon in F_6_ pigs. Violin plots show that the richness and diversity of fecal microbiota in castrated boars were closer to gilts. **(B,C)** The differential bacterial taxa before and after castration at the family and genus level. **(D)** The sex-biased bacterial genera between entire boars and gilt counterparts were no longer the distinct bacterial genera between castrated boars and gilts. **(E)** The top 10 biomarkers of OTUs that could discriminate the samples from entire or castrated boars by Random Forest model. Biomarker OTUs were ranked in descending order of importance to the accuracy of the model. **(F)** Receiver operating curve (ROC) for entire and castrated boars. The AUC value was 98.85% with 95% CI of 97.08–100%.

Intriguingly, compared to gilts, entire boars showed significantly lower α-diversity of gut microbiota. To determine whether androgens could significantly affect the gut microbial composition of the entire boars, the phylogenetic composition of gut microbiota was compared before and after castration. Significant differences in the gut microbial community structures were observed between samples harvested before and after castration (ANOSIM, *R* = 0.631, *P* = 1.00E-03, Figure 2B). Compared to entire boars, the α-diversity indices of fecal microbiota measured as Shannon and Observed species significantly increased in castrated boars (*P* = 4.00E-03 and 1.17E-05, respectively) (Figure 3A), but only the index of Observed species significantly differed between castrated boars and gilt counterparts (*P* = 9.82E-04) (Figure 3A). At the taxonomic level, 12 families and 13 genera were detected with significantly different abundances before and after castration (Figure 3B,C). Eight bacterial genera previously identified as sex-biased bacteria between entire boars and gilts showed no difference in their abundance between castrated boars and gilts, including five genera showing differential abundance before and after castration (Figure 3D).

A total of 145 OTUs showed significantly discriminating abundances in feces before and after castration (Supplementary Table S3). Interestingly, similar to the sex-biased OTUs identified between entire boars and gilts, these OTUs mainly belonged to Ruminococcaceae, Lachnospiraceae, Veillonellaceae, Christensenellaceae, *Prevotella*, *Coprococcus*, *Oscillospira*, *Treponema*, *Faecalibacterium*, *Blautia*, and *Ruminococcus*. A significantly lower number of sex-biased OTUs was identified between castrated boars and gilts (42 OTUs; Supplementary Table S4). Ruminococcaceae, *Blautia*, *Oscillospira*, *Prevotella*, and *Treponema* remained the sex-biased bacterial taxa between castrated boars and gilts, whilst Lachnospiraceae, Veillonellaceae, Christensenellaceae, *Coprococcus* and *Faecalibacterium* no longer represented sex-biased bacterial taxa (Supplementary Table S4). Random forest analysis identified 10 OTUs primarily annotated to *Treponema*, *Ruminococcus*, and Clostridiaceae, which could be used to discriminate uncastrated boars from castrated boars with a diagnostic accuracy of AUC 98.85% (Figure 3E,F).

### Sex-Biased Functional Capacity of the Gut Microbiome and Its Relationship With Sex-Biased Bacteria

To identify KEGG pathways of the gut microbiome that are influenced by host gender, the potential functional capacity of gut microbiome was predicted based on 16S rRNA gene sequences. In Duroc pigs, the KEGG pathways of Transporters and ATP-binding cassette (ABC) transporters were enriched in gilts (Supplementary Table S5). Spearman correlation analysis found that *Treponema* and *Bulleidia* positively correlated with ABC transporter function (Supplementary Figure S2A). In the F_6_ pig population, KEGG pathways including bacterial chemotaxis, the two-component system, flagellar assembly, fatty acid metabolism, and bacterial motility were enriched in gilts, whilst peptidases, purine metabolism, galactose metabolism, and starch and sucrose metabolism, which were related to protein and carbohydrate metabolism, were enriched in entire boars (Supplementary Table S5). *Treponema* was positively associated with fatty acid metabolism, flagellar assembly, the two-component system and bacterial motility, whilst *Prevotella* and *Sutterella* were negatively correlated with these pathways. *Ruminococcus*, *Eubacterium*, *Faecalibacterium*, and *Blautia* which were enriched in entire boars, positively correlated with the functional pathways which were of higher abundance in entire boars (Supplementary Figure S2B).

Differential KEGG pathways before and after castration were similar to those of entire boars and gilts in the F_6_ population. The gut microbiome after castration was enriched in pathways related to bacterial motility, the two-component system, bacterial chemotaxis, flagellar assembly, and fatty acid and propanoate metabolism, whilst pathways related to carbohydrate metabolism, peptidases and DNA replication were enriched in entire boars (Supplementary Table S5). *Treponema* was positively associated with flagellar assembly, bacterial chemotaxis and bacterial motility, but negatively correlated to the pathways enriched in entire boars. *Prevotella* and *Sutterella* negatively correlated with the KEGG pathways enriched in the gut microbiome after castration. Additionally, *Roseburia*, *Faecalibacterium*, and *Catenibacterium* were positively related to the KEGG pathways enriched in entire boars (Supplementary Figure S2C). Interestingly, although some KEGG pathways showed differential abundances between castrated boars and their gilt counterparts, all these pathways did not achieve significance level after corrected the multiple testing.

### Differential Metabolite Profiles Between Gilts and Entire Boars, and Their Correlation to Sex-Biased Bacterial Taxa

**FIGURE 4:**
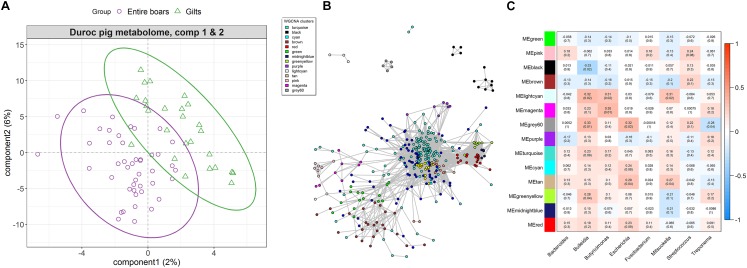
The differentiation of host serum metabolite profile between entire boars and gilts, and its associations with sex-biased bacteria in the Duroc pig cohort. **(A)** sPLS-DA plot of serum metabolite profiles, which indicates the significant differentiation of serum metabolite profiles between entire boars and gilts. **(B)** Co-occurrence network of serum metabolite features. The metabolites (nodes) are colored according to WGCNA module colors. Only those correlations with | *r*| > 0.2 between two edges were presented. **(C)** Correlations between metabolite modules and sex-biased bacteria. Each box of the matrix represents the correlation between one metabolite module and a sex-biased bacterial taxon at the genus level. The correlation coefficient (r) and the corresponding *P*-value (in brackets) were filled in the small boxes. The color gradient represents the values of correlation coefficients (red for positive correlations and blue for negative correlations).

To systematically evaluate shifts in the host serum metabolome between entire boars and gilts, the serum metabolomic profiles were determined using UPLC-MS in Duroc pigs. After normalization, a total of 2,347 endogenous metabolites were obtained. An obvious shift in the global metabolome was observed between entire boars and gilts (Figure 4A). Specifically, a total of 8 metabolite features were identified as sex-biased (*P* < 0.001, FDR <0.2). These features were mainly annotated to putative metabolites related to androgen and cresol metabolism according to the HMDB database. Excluding p-Cresol sulfate and o/p/m-Cresol that were enriched in gilts, entire boars had a higher abundance of sex-biased metabolites (Table 1).

**Table 1:** Metabolites showing distinct abundance between entire boars and gilts.

Retention time (RT)–m/z	Gilts (mean ± SD)	Entire boars (mean ± SD)	*P*-value	*Q*-value	HMDB	Putative compound
6.66_449.2542 m/z	6.114 ± 24.436	136.364 ± 164.545	2.61E-08	3.06E-05	HMDB10321	3,17-androstanediol glucuronide
					HMDB10359	17-hydroxyandrostane-3-glucuronide
					HMDB10339	3-alpha-androstanediol glucuronide
4.88_369.1737 m/z	5.989 ± 22.735	106.036 ± 119.298	5.65E-08	4.42E-05	HMDB02759	Androsterone sulfate
					HMDB06278	5a-dihydrotestosterone sulfate
4.78_367.1578 m/z	9.194 ± 35.349	94.333 ± 109.472	5.95E-07	3.49E-04	HMDB02833	Testosterone sulfate
					HMDB01032	DHEA sulfate
5.13_465.2462 m/z	2.763 ± 12.00	41.119 ± 52.68	2.94E-06	1.38E-03	HMDB10365	3-alpha-hydroxy-5-alpha-androstane-17-one 3-D-glucuronide
					HMDB06203	5-alpha-Dihydrotestosterone glucuronide
					HMDB04484	Etiocholanolone glucuronide
					HMDB02829	Androsterone glucuronide
4.61_465.2492 m/z	6.665 ± 17.311	41.547 ± 50.14	1.96E-04	7.54E-02	HMDB10365	3-alpha-hydroxy-5-alpha-androstane-17-one 3-D-glucuronide
					HMDB06203	5-alpha-Dihydrotestosterone glucuronide
					HMDB04484	Etiocholanolone glucuronide
					HMDB02829	Androsterone glucuronide
3.64_187.0070 m/z	1895.955 ± 1301.196	846.586 ± 604.482	4.06E-04	0.12	HMDB11635	p-Cresol sulfate
18.19_467.2971 m/z	481.362 ± 26.737	504.372 ± 33.514	7.58E-04	0.17	HMDB32521	Stearyl citrate
					HMDB39225	2-Stearyl citrate
3.64_107.0504 m/z	109.690 ± 76.958	48.810 ± 37.095	7.80E-04	0.17	HMDB02055	o-Cresol
					HMDB01858	p-Cresol
					HMDB02048	m-Cresol

The correlation between sex-biased bacteria and host serum metabolites was then analyzed using all 2,347 metabolite features. Co-abundance networks among metabolites were constructed based on the log_10_ transformed values of the serum metabolome profiles. Different colors were used to represent distinct modules within the networks. As the result, turquoise and brown metabolic modules were the largest, containing 335 and 34 metabolite features, respectively (Figure 4B). Metabolites from the same metabolic pathway should be preferentially clustered into the same module. For instance, the metabolite features related to androgen metabolism were clustered into the module grey60, and the greenyellow module that primarily contained metabolites related to bile acid metabolism (Supplementary Table S6). Several significant correlations were identified between metabolite modules and sex-biased bacterial genera (Figure 4C). The grey60 module comprising the sex-biased metabolite features was annotated to testosterone sulfate, androsterone sulfate, 3,17-androstanediol glucuronide and 3-alpha-androstanediol glucuronide. These showed a positive correlation with *Bulleidia* and *Escherichia*, but were negatively correlated to *Treponema*. The greenyellow module consisted of metabolite features mainly comprising primary and secondary bile acid metabolism that positively correlated with *Bulleidia* (Figure 4C). To further confirm these relationships, correlation analysis was independently performed between the sex-biased bacterial genera and the metabolite features within grey60 and greenyellow modules. The same significant correlations were observed between *Treponema*, *Bulleidia*, and *Escherichia* and each metabolite featured in the grey60 module (Supplementary Figure S3). The same correlation was also observed between *Bulleidia* and each metabolite feature within the greenyellow module, irrespective of some correlations are not statistically significant (Supplementary Figure S4).

## Discussion

In this study, the effect of host gender on gut microbial composition was systematically evaluated through a comparison of the fecal microbiota communities between entire boars and gilts, and gonadectomy on entire boars to further investigate the influence of androgen on the microbial composition. In addition, the correlation of sex-biased bacteria with predicted KEGG pathways of the gut microbiome and host serum metabolites was investigated. To our knowledge, this is one of only a handful of studies that have evaluated the interaction between sex-biased bacteria and host metabolism.

Consistent with our previous findings in mice and humans that indicated a lower level of gut microbial diversity in males (Yurkovetskiy et al., 2013; Elderman et al., 2018; Gao et al., 2018), entire boars had a lower diversity of fecal microbiota when compared to gilts in both tested and validated pig populations. This suggested an influence of sex hormones, particularly androgen, in gut microbial composition. PCoA analysis also revealed a large variation in gut microbial composition between entire boars and gilts (Figure 1A, 2A). However, Han et al. (2018) reported that the intestinal microbial composition of pigs was differed according to gender. This discrepancy could be caused by the variable age of the pigs and small sample size used in that study (Han et al., 2018).

Many of the sex-biased bacteria identified in this study have been reported in both mice and humans. For example, Veillonellaceae, Enterobacteriaceae, *Lactobacillus*, *Roseburia*, *Eubacterium*, *Sutterella*, and *Coprococcus* represent sex-biased bacteria, previously been reported in mice (Yurkovetskiy et al., 2013; Org et al., 2016; Wang et al., 2016; Elderman et al., 2018); *Eubacterium*, *Blautia*, and *Treponema* also represent sex-biased bacteria in humans (Schnorr et al., 2014). Furthermore, *Ruminococcus*, *Eubacterium*, and *Streptococcus* have been previously reported as sex-biased bacteria in swine (Zhou et al., 2015). Estrogen inhibits the unregulated growth of *Escherichia coli* in the intestine of rats (Yang et al., 2018). In this study, *Escherichia* displayed the highest abundance in entire boars. These results provided further evidence to support the sex effect of the bacterial taxa. Several sex-biased bacteria were also identified, including *Faecalibacterium*, *Catenibacterium*, and Christensenellaceae. Some of these bacterial taxa were reported as sex-biased only in tested and/or validated pig populations. This discrepancy may be caused by different diets, farm conditions and host genetics.

Previous studies demonstrated that, compared to entire males, the composition of the gut microbiota in castrated male mice is comparable to that of female mice (Yurkovetskiy et al., 2013; Han et al., 2018). Consistent with these findings, gonadectomy significantly shaped the fecal microbial composition of pigs. The gut microbial structure of castrated boars was of higher similarity to gilts (no significant differences in the Shannon index between castrated boars and gilts) and the abundance of 13 bacterial genera dramatically shifted following castration (Figure 3C). These taxa are likely to be influenced by the host androgen (testosterone) levels. As an example, *Bacteroides* was of significantly higher abundance in entire boars in F_6_ population, and recently shown to positively correlate with testosterone through bi-directional cross-talk (Liu et al., 2017b).

The KEGG pathways of the gut microbiome enriched in gilts were related to cell motility (flagellar assembly) and membrane transport (two-component system and ABC-transporters), which have recently been shown to promote high fat levels in pigs, obese mice, and humans (Turnbaugh et al., 2006, 2009; Duca et al., 2014; Yang et al., 2016; Hou et al., 2017). In particular, the two-component system is involved in signal transduction (Alm et al., 2006; Cui et al., 2011) and regarded as a metabolic reaction center that couples nutrient sensing to the dynamic regulation of monosaccharide metabolism (Sonnenburg et al., 2006). In addition, ABC transporters mediate the uptake of a large variety of nutrients, and regulate the export of lipids, and primary and secondary metabolites (Maruyama et al., 2015; Hou et al., 2017). These KEGG pathways were also enriched in pigs with a low feed efficiency (i.e., those who have more fat accumulation) indicating that a higher ratio of feed intake is necessary to ensure body weight (muscle mass increase) gain (Wenk et al., 1980; Yang et al., 2017). It is known that sows exhibit lower feed efficiency than boars. The over-representation of flagellar assembly, the two-component system and ABC transporters in the gut microbiome of gilts, and their synergistic correlation with *Treponema* may contribute to increased energy absorption, leading to host fat deposition that lowers the feed efficiency of gilts. However, pathways related to peptidases and carbohydrate metabolism were enriched in entire boars, and positively associated with *Blautia*, *Faecalibacterium*, *Ruminococcus*, *Eubacterium*, and *Catenibacterium*. *Blautia*, *Eubacterium*, and *Faecalibacterium* produce butyric acid that improves intestinal development and health through its anti-inflammatory properties (Kanauchi et al., 2006; Louis et al., 2010, 2014; Jenq et al., 2015; Breyner et al., 2017; Chen et al., 2017). Moreover, *Eubacterium*, *Faecalibacterium*, and *Catenibacterium* promote carbohydrate utilization and fermentation (Freier et al., 1994; Kageyama and Benno, 2000; Duncan et al., 2002), and *Ruminococcus* has dipeptidyl peptidase activity (Wallace et al., 1997). As such, the increased abundance of these KEGG pathways and the corresponding bacterial taxa in the gut microbiome of boars may promote the utilization of dietary polysaccharides and proteins in entire boars, improving their feed efficiency (Yang et al., 2017).

Correlation analysis established a relationship between the shifts in host serum metabolites and the changes in gut bacteria. For instance, a negative correlation was found between the metabolites related to androgen metabolism and *Treponema*. This result was supported by reports demonstrating that higher levels of testosterone can inhibit *Treponema* growth (Clark and Soory, 2006; Garcia-Gomez et al., 2013). A positive correlation between androgen-related metabolites and *Escherichia* was also supported by previous studies that indicated that β-glucuronidase from *E. coli* cleaves glucuronide from androgen conjugates, releasing free androgens for re-absorption (Graef et al., 1977). *E. coli* has also been shown to upregulate host testosterone levels and cooperatively enhance androgen-dependent protection in Type 1 diabetes (An and Kasper, 2013). These results suggested that interactions between androgens and the gut microbiota may influence the host phenotypes, such as porcine fat deposition and feed efficiency.

## Conclusion

In conclusion, this study detected an obvious effect of host gender on fecal microbiota structure, and identified numerous sex-biased bacterial taxa. The results also demonstrate that gonadectomy significantly shifts the fecal microbiota composition of boars toward that of gilts. The gut microbiome of entire boars had a greater ability to utilize dietary carbohydrate and proteins, whilst the gut microbiome of gilts promotes energy harvesting. The correlation between sex-biased bacteria (*Treponema*, *Escherichia*, and *Bulleidia*) and androgen-related metabolites suggested a significant interaction between the gut microbiota and host sex hormones. Taken together, these results provide important insights into the interaction between the gut microbiome and host serum metabolites that enhances our knowledge of the interaction between host gender and the gut microbiota. This information can be used to understand the influence of these parameters on host metabolism and phenotypes, including the growth and fat levels of pigs, which is informative for the pig industry. Furthermore, estrogen should also play an important role in host gut microbial composition and metabolism. It would be interesting in the future exploring the effects of gonadectomy also in females to detect possible estrogen-mediated effects on the host microbial composition, protein profiles and metabolism.

## Author Contributions

LH conceived and designed the experiments and revised the manuscript. CC conceived and designed the experiments, analyzed the data, and wrote and revised the manuscript. MH performed the experiments, analyzed the data, and wrote the manuscript. JG performed the experiments. JW, YZ, HF, HY, and SK collected the samples and extracted the DNA. All authors read and approved the final manuscript.

## Conflict of Interest Statement

The authors declare that the research was conducted in the absence of any commercial or financial relationships that could be construed as a potential conflict of interest.

## Footnotes

1 http://www.metaboanalyst.ca/

## Supplementary Material

FIGURE S1 Distinct gut microbial compositions between entire boars and gilts in the F_6_ pig cohort. **(A)** Comparison of α-diversity of the fecal microbiota (observed species and Shannon index) between gilts and entire boars. Violin plots show that gilts had the higher richness and diversity of fecal microbiota than entire boars. ^∗^*P* < 0.05, ^∗∗^*P* < 0.01, and ^∗∗∗^*P* < 0.001. **(B,C)** The sex-biased bacterial taxa at the family and genus level. **(D)** The top 10 biomarkers of OTUs that could discriminate the male and female samples by Random Forest model. Biomarker OTUs were ranked in descending order of importance to the accuracy of the model. **(E)** Receiver operating curve (ROC) for the Duroc population. The AUC was 94.54% with the 95% CI of 88.5–100%.

FIGURE S2 Correlation between the sex-biased KEGG functional pathways and the sex-biased fecal bacteria. **(A)** Heatmap of the Spearman’s rank correlation coefficient between two KEGG pathways and 15 sex-biased fecal bacteria in the Duroc pig cohort. **(B)** Correlation between 17 sex-biased KEGG pathways and 16 sex-biased bacteria in F_6_ pigs. **(C)** Correlation between 25 differential KEGG pathways and 13 differential bacterial taxa between entire boars and castrated boars. ^∗^*P* < 0.05, ^∗∗^*P* < 0.01, and ^∗∗∗^*P* < 0.001.

FIGURE S3 Correlation between sex-biased bacterial taxa and metabolite features. The figures indicate the correlation between relative abundances of *Treponema* **(A)**, *Bulleidia* **(B)**, and *Escherichia* **(C)**, and serum concentration of each metabolite in metabolic module grey60.

FIGURE S4 Spearman correlation between the relative abundance of *Bulleidia* and the serum concentration of each metabolite feature in the metabolic module greenyellow.

TABLE S1 The sex-biased OTUs in the Duroc population.

TABLE S2 The sex-biased OTUs in the F_6_ population.

TABLE S3 The differential OTUs between entire boars and castrated boars in the F_6_ population.

TABLE S4 The differential OTUs between castrated boars and gilt counterparts in the F_6_ population.

TABLE S5 Differential KEGG pathways between entire boars and gilts in Duroc pigs, and between entire boars and castrated boars in F6 pig population.

TABLE S6 Metabolite modules of serum compounds *via* WGCNA method in the Duroc pig population.
