# Supplementary figures and images for: Host Gender and Androgen Levels Regulate Gut Bacterial Taxa in Pigs Leading to Sex-Biased Serum Metabolite Profiles
Source: Front Microbiol. 2019 Jun 18;10:1359. doi: 10.3389/fmicb.2019.01359 (PMC6591444; doi:10.3389/fmicb.2019.01359)

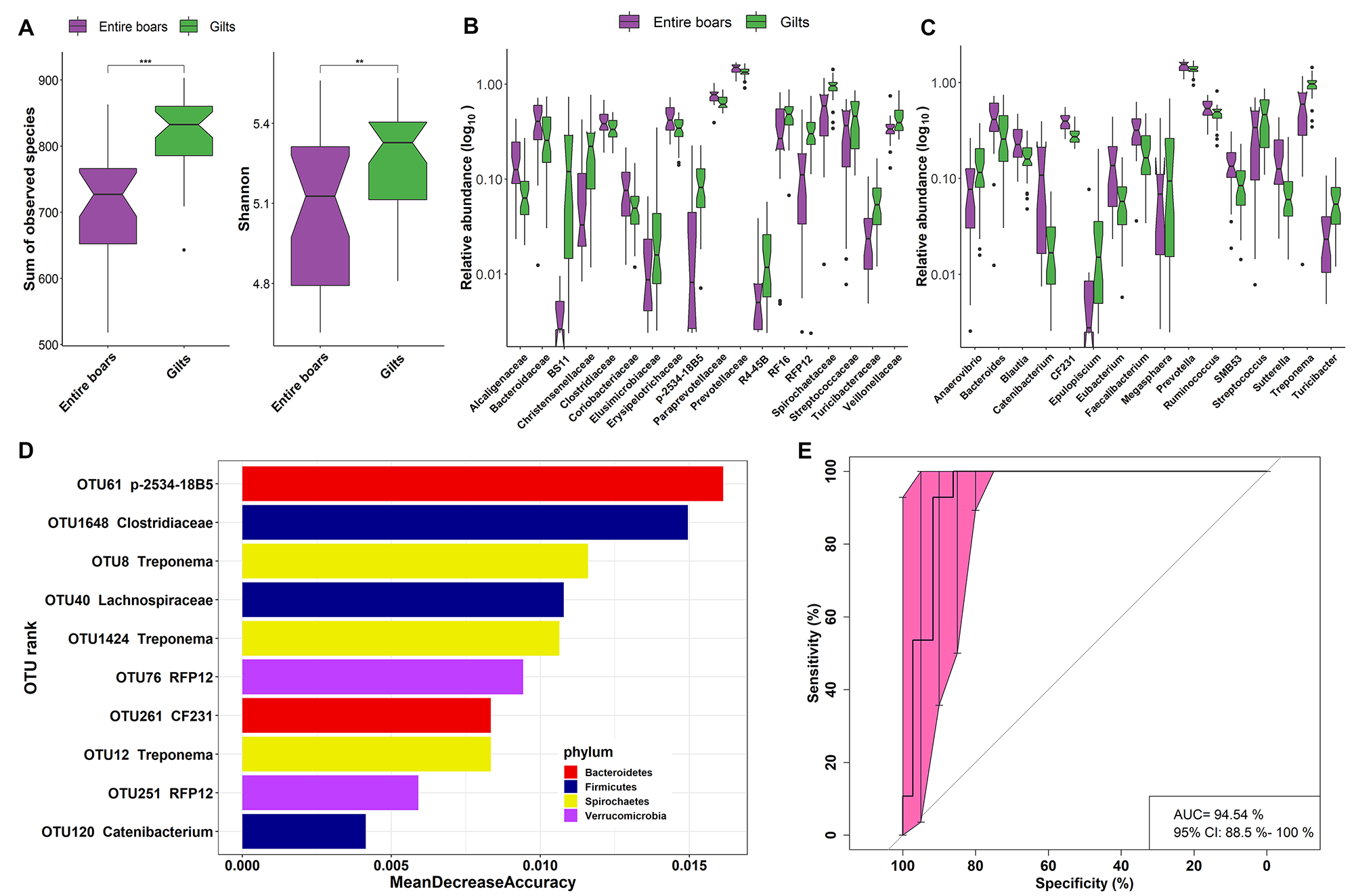

Supplement: FIGURE S1 — Distinct gut microbial compositions between entire boars and gilts in the F6 pig cohort. (A) Comparison of α-diversity of the fecal microbiota (observed species and Shannon index) between gilts and entire boars. Violin plots show that gilts had the higher richness and diversity of fecal microbiota than entire boars. ∗P < 0.05, ∗∗P < 0.01, and ∗∗∗P < 0.001. (B,C) The sex-biased bacterial taxa at the family and genus level. (D) The top 10 biomarkers of OTUs that could discriminate the male and female samples by Random Forest model. Biomarker OTUs were ranked in descending order of importance to the accuracy of the model. (E) Receiver operating curve (ROC) for the Duroc population. The AUC was 94.54% with the 95% CI of 88.5–100%. [file Image_1.TIF]

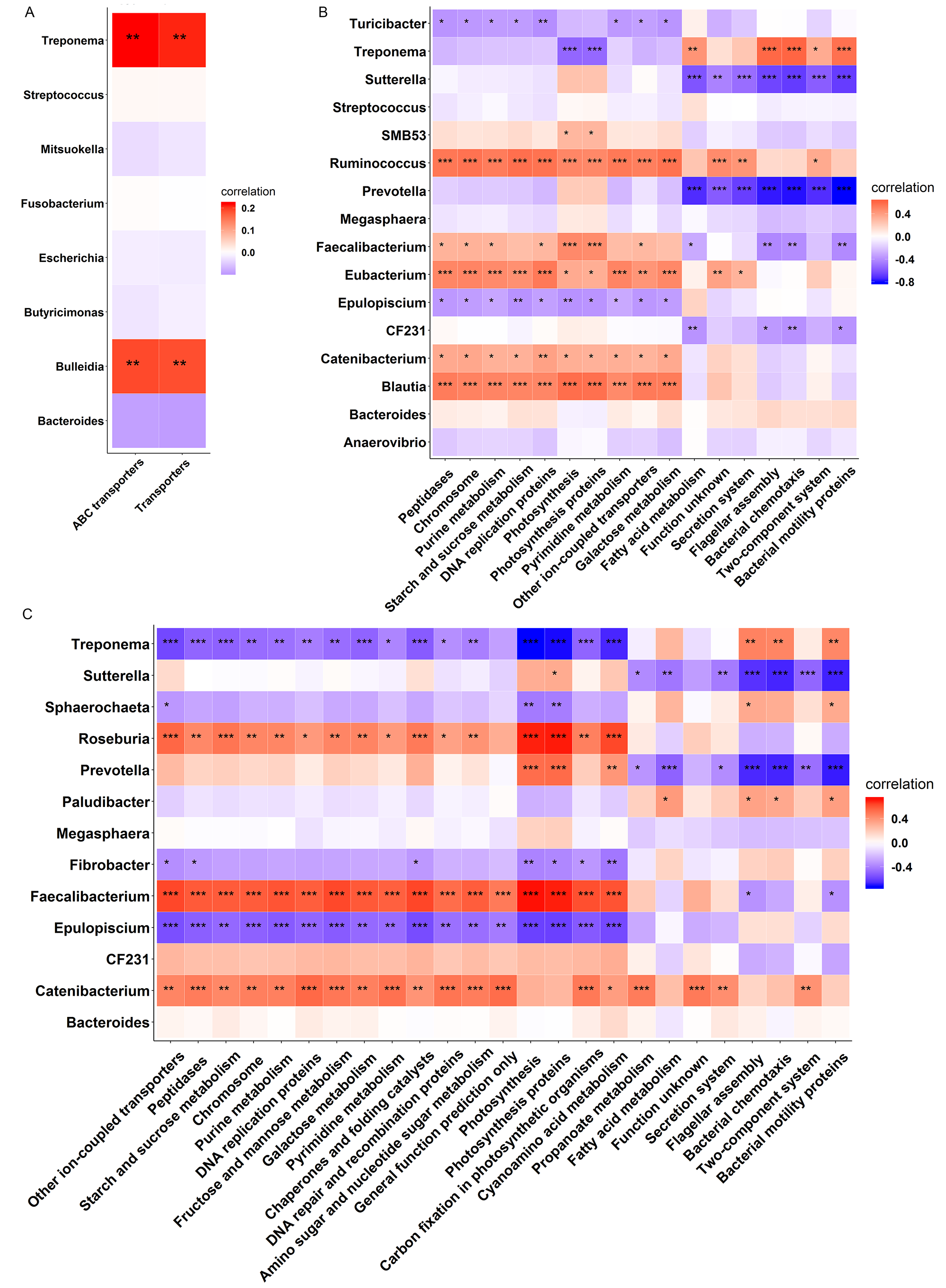

Supplement: FIGURE S2 — Correlation between the sex-biased KEGG functional pathways and the sex-biased fecal bacteria. (A) Heatmap of the Spearman’s rank correlation coefficient between two KEGG pathways and 15 sex-biased fecal bacteria in the Duroc pig cohort. (B) Correlation between 17 sex-biased KEGG pathways and 16 sex-biased bacteria in F6 pigs. (C) Correlation between 25 differential KEGG pathways and 13 differential bacterial taxa between entire boars and castrated boars. ∗P < 0.05, ∗∗P < 0.01, and ∗∗∗P < 0.001. [file Image_2.TIF]

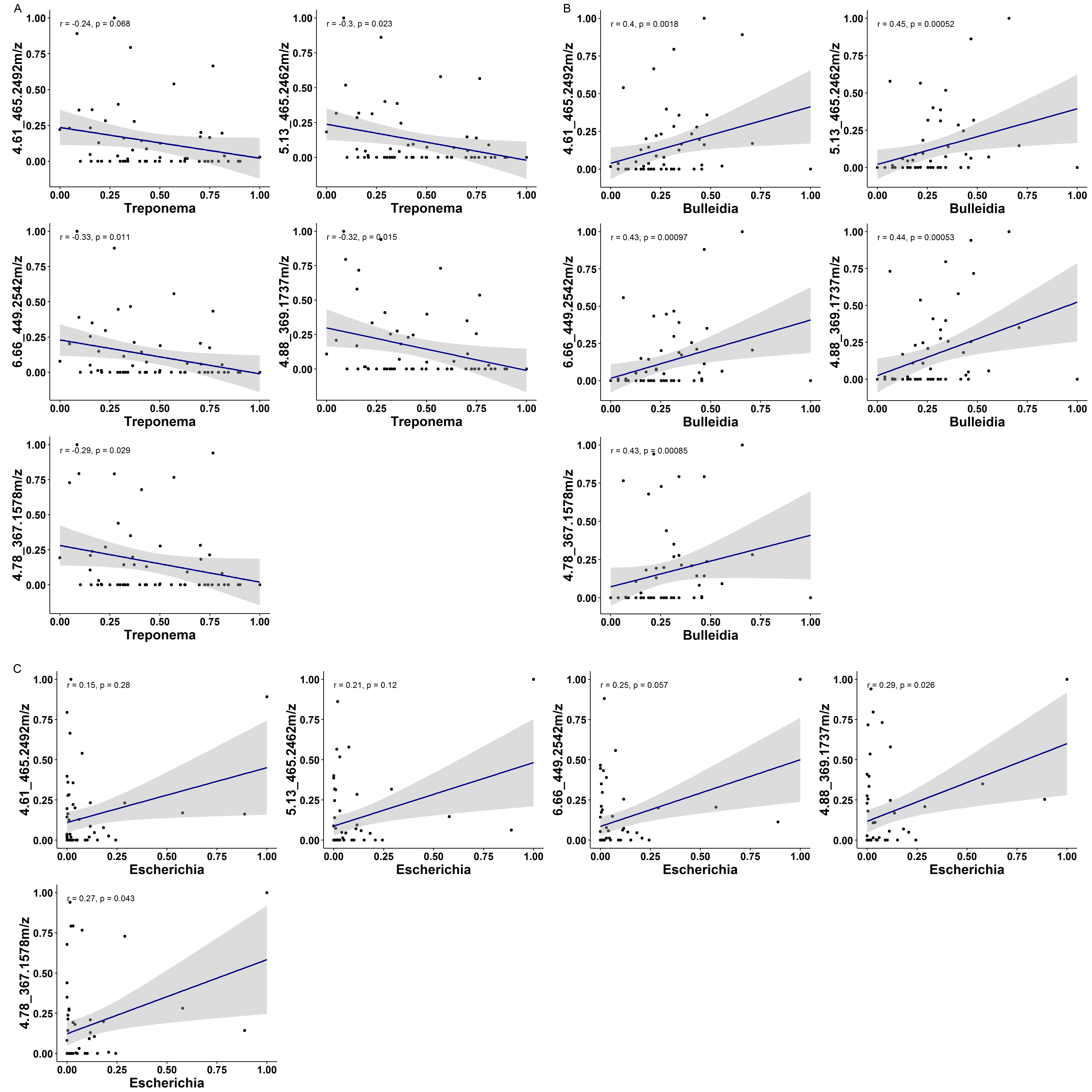

Supplement: FIGURE S3 — Correlation between sex-biased bacterial taxa and metabolite features. The figures indicate the correlation between relative abundances of Treponema (A), Bulleidia (B), and Escherichia (C), and serum concentration of each metabolite in metabolic module grey60. [file Image_3.TIF]

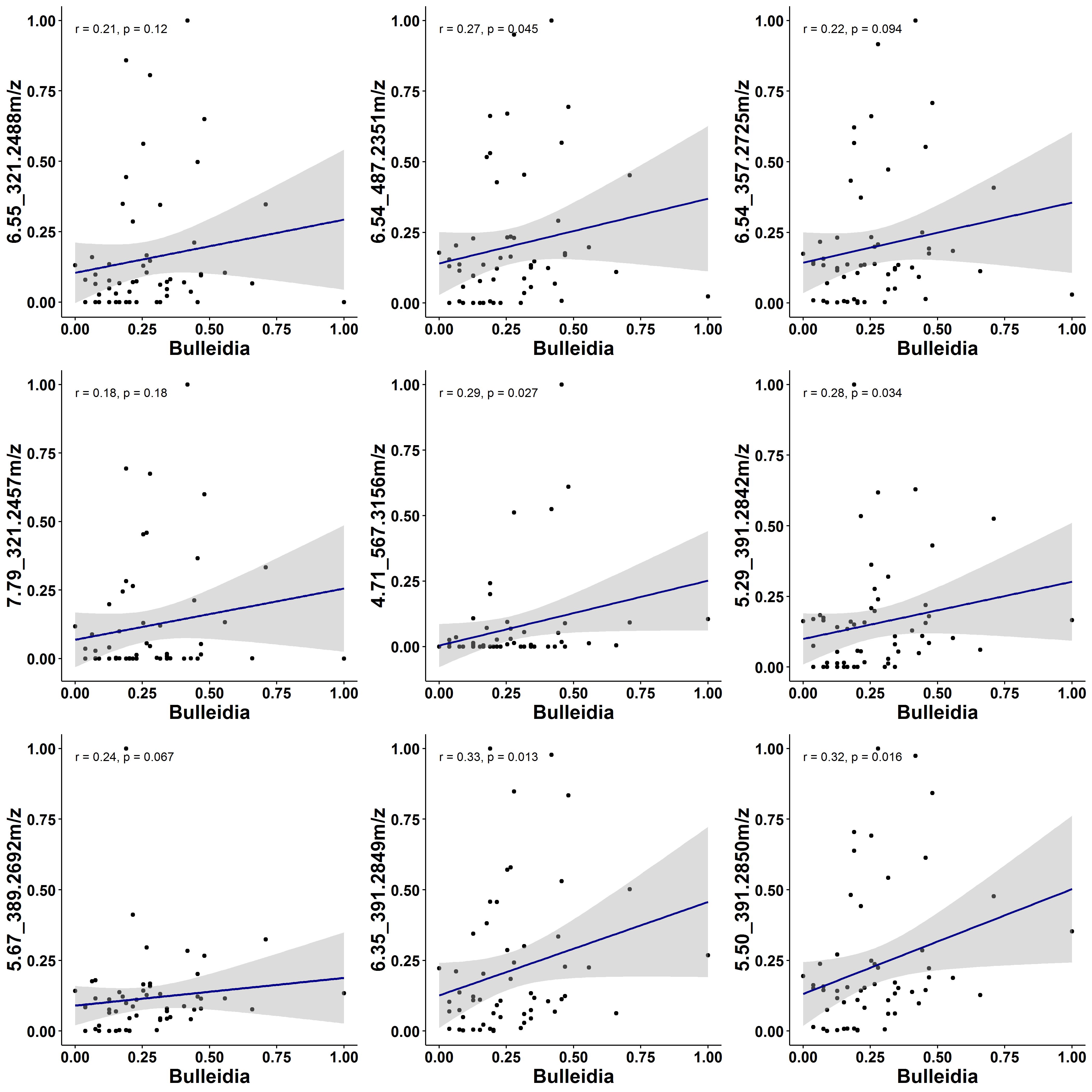

Supplement: FIGURE S4 — Spearman correlation between the relative abundance of Bulleidia and the serum concentration of each metabolite feature in the metabolic module greenyellow. [file Image_4.TIF]
